# Detectable land use impact on methanotrophs and methanogens in kettle hole sediments but not on net methane production potentials

**DOI:** 10.1093/femsec/fiaf050

**Published:** 2025-05-01

**Authors:** Danica Kynast, Florian Reverey, Lars Ganzert, Hans-Peter Grossart, Gunnar Lischeid, Steffen Kolb

**Affiliations:** Leibniz Centre for Agricultural Landscape Research, Working Group: Microbial Biogeochemistry, Eberswalder Str. 84, D-15374 Müncheberg, Germany; Humboldt University of Berlin, Albrecht Daniel Thaer Institute of Life Sciences, Invalidenstraße 42, D-10115 Berlin, Germany; Leibniz Centre for Agricultural Landscape Research, Working Group: Dimensionality Assessment and Reduction, Eberswalder Str. 84, D-15374 Müncheberg, Germany; Leibniz Institute of Freshwater Ecology and Inland Fisheries, Department of Plankton and Microbial Ecology, Alte Fischerhütte 2, OT Neuglobsow, D-16775 Stechlin, Germany; Leibniz Institute of Freshwater Ecology and Inland Fisheries, Department of Plankton and Microbial Ecology, Alte Fischerhütte 2, OT Neuglobsow, D-16775 Stechlin, Germany; Potsdam University, Institute for Biochemistry and Biology, Maulbeerallee 2, D-14469 Potsdam, Germany; Leibniz Centre for Agricultural Landscape Research, Working Group: Dimensionality Assessment and Reduction, Eberswalder Str. 84, D-15374 Müncheberg, Germany; Potsdam University, Institute of Environmental Science and Geography, Karl-Liebknecht-Str. 24–25, D-14476 Potsdam, Germany; Leibniz Centre for Agricultural Landscape Research, Working Group: Microbial Biogeochemistry, Eberswalder Str. 84, D-15374 Müncheberg, Germany; Humboldt University of Berlin, Albrecht Daniel Thaer Institute of Life Sciences, Invalidenstraße 42, D-10115 Berlin, Germany

**Keywords:** kettle hole, land use, methane, methanogens, methanotrophs, sediments

## Abstract

Kettle holes (KHs) are dynamic freshwater systems and potential sources of the greenhouse gas methane. Due to their small size (<1 hectare), KHs are subject to inorganic and organic matter input from their terrestrial surroundings, governed by land use. Matter inputs include inorganic solutes that are alternative electron acceptors and impact on methanotrophs and methanogens. Thus, they might affect methane net production. We sampled 10 kettle hole sediments embedded in landscapes with either agricultural or forest land use and determined their (i) potential net methane production rates, (ii) the composition of their microbial communities, and (iii) physicochemical soil parameters. Potential net methane production did not significantly differ by land use type but between single KHs. However, land use type had a strong impact on methanotroph and methanogen and on total bacterial and archaeal microbiota structure. Relative abundances of methanotrophs and methanogens were significantly higher in agricultural KHs, and their relative abundances were among the most influential variables projecting net methane production potentials along with nutrient status and water content. Land use type was thus identified as a major factor that impacts the structure and biodiversity of general and methane-cycling microbiota, but it did not affect net methane production potentials.

## Teaser

The land use type around kettle holes, small, dynamic freshwater systems, impacts the general and methane-cycling microbiota composition in the sediments, but does not affect the potential net methane production rates.

## Introduction

Methane (CH_4_) is the second most important greenhouse gas (Kirschke et al. 2013, Jackson et al. 2024) with a 100-year global warming potential 28 times higher than CO_2_ (Saunois et al. 2020). CH_4_ concentrations in the atmosphere increased by 156% since the eighteenth century, which is attributable to human activities (IPCC 2021). In addition to anthropogenic sources, natural sources include most notably wetlands, but also other freshwater systems such as lakes and small ponds, for which still much uncertainty on their contribution to the atmospheric methane burden exists (Saunois et al. 2020). An important factor impacting CH_4_ emissions of lakes is eutrophication, which is projected to increase CH_4_ emissions substantially over the next hundred years (Beaulieu et al. 2019, Rosentreter et al. 2021).

A type of freshwater environment, in particular prone to eutrophic conditions, is water-filled kettle holes (KH), also known as pot holes. They are dynamic freshwater systems with water bodies of a maximum of 1 ha and often located within landscapes of intense agricultural use. KHs are landscape elements usually considered to be classified in between wetlands and ponds (Kalettka and Rudat 2006) and can be net emitters of greenhouse gasses including CH_4_ (Merbach et al. 2002, Reverey et al. 2018). KHs exist in young moraine landscapes and were created by delayed melting of ice blocks broken off from glaciers during their retreat (Kalettka 1996). Their hydroperiods are often very diverse (Kalettka and Rudat 2006). Despite their often small surface area, KHs are highly abundant in certain areas and have been described in North America, North Asia, northern Europe, as well as in New Zealand in the southern hemisphere (Vasić et al. 2020). The Uckermark region in northeastern Brandenburg (Germany) features KH abundances up to 40 KHs per km^2^ (Kalettka and Rudat 2006), making them a considerable part of the landscape (Premke et al. 2016).

Most KHs in the area are considered eutrophic (Lischeid et al. 2018). Due to their size and location usually in depressions of rolling hill landscapes, they are subject to inorganic and organic matter input from their surroundings. This input can include soil particles and fertilizer, as well as plant debris mainly depending on land use practices in the surrounding area (Nitzsche et al. 2017). Land use around the KH also impacts carbon (C)- and nitrogen (N)-cycling processes in KHs (Nitzsche et al. 2017, Reverey et al. 2018), whereby anoxic conditions are more prevalent in forest KHs since reduced light intensity is often limiting photosynthesis (Nitzsche et al. 2017, Bizic et al. 2022). Furthermore, land use shapes the active aquatic community in the KH, which responds to nutrient and organic matter input (Bizic et al. 2022). Therefore, an impact of land use on CH_4_-cycling microorganisms is likely, and this has the potential to affect CH_4_ net emissions from these landscape elements (Reverey et al. 2018, 2021).

In particular, biogenic CH_4_ production and consumption in these environments are largely mediated by microorganisms, so-called methanogens and methanotrophs, respectively. The generally accepted concept is that methanogenic archaea produce CH_4_ in anoxic zones of a terrestrial or aquatic environment, while methanotrophic bacteria oxidize it to CO_2_ under oxic conditions (Knief 2019). Accordingly, CH_4_ is produced under anoxic conditions as the final step of the breakdown of organic matter (Conrad 1996, Drake et al. 2009). Methanogens can be characterized by their respective CH_4_ production mechanism as (i) acetoclastic, i.e. the utilization of acetate for CH_4_ formation, (ii) hydrogenotrophic, i.e. the utilization of H_2_ and CO_2_, and (iii) methylotrophic, i.e. the disproportionation of various methylated compounds to form CH_4_ (Conrad 2020). Hydrogenotrophic and acetoclastic methanogenesis are generally considered as most important for CH_4_ production (Knief 2019). However, substantial contribution of methylotrophic methanogenesis has recently been proven in certain freshwater environments such as thermokarst lake sediments, where it can even represent the dominant CH_4_ formation pathway (Yang et al. 2023).

The biological oxidation of CH_4_ is considered the largest global terrestrial CH_4_ sink and is mediated mostly by methanotrophs (Saunois et al. 2020). These aerobic Bacteria belong to *Alphaproteobacteria, Gammaproteobacteria, Verrucomicrobia* (Semrau et al. 2010, Guerrero-Cruz et al. 2021), and *Actinomycetia*. In the latter order, methanotrophy was only newly discovered in the family *Mycobacteriaceae* (van Spanning et al. 2022). While methanotrophs of *Verrucomicrobia* and *Actinomycetia* comprise extremophilic species that prefer low pH and high temperatures, the methanotroph species belonging to *Alphaproteobacteria* and *Gammaproteobacteria* play a large role in reducing global CH_4_ emissions. They act usually as a filter at the oxic-anoxic interface, where they have access to both the CH_4_ produced at anoxic areas and molecular oxygen required as a terminal electron acceptor for CH_4_ oxidation (Hanson and Hanson 1996, Knief 2019, Thottathil et al. 2022). However, some aerobic methanotrophs, in particular gammaproteobacterial ones, can be even active under supposedly non-optimal conditions such as anoxia in lake water columns (van Grinsven et al. 2020, Oswald et al. 2016) and lake sediments (Martinez-Cruz et al. 2017, He et al. 2012, Lyautey et al. 2021, Bar-Or et al. 2017). These observations significantly broaden the expected environmental niche for biological CH_4_ oxidation. Under these conditions, they can support CH_4_ oxidation via symbiosis with phototrophic organisms by directly consuming the phytosynthesis-drived molecular oxygen (Milucka et al. 2015, Oswald et al. 2015). In addition to oxygen-dependent CH_4_ oxidizers, some microorganisms are capable of utilizing alternative electron acceptors to conduct anaerobic oxidation of CH_4_ (AOM). AOM is known to impact CH_4_ emissions from marine sediments (Kevorkian et al. 2022) and freshwater wetlands (Segarra et al. 2015). Known compounds used for this process include sulfate, nitrate, nitrite, ferric iron, oxidized manganese, and even humic substances (Cui et al. 2015, Bar-Or et al. 2017, Kits et al. 2015, Zheng et al. 2020). Nitrite-dependent AOM is conducted by *Candidatus* Methylomirabilis oxyfera-like members belonging to the phylum *Methylomirabilota* (formerly known as NC10 group). *Ca*. Methylomirabilis oxyfera conducts oxygen-dependent oxidation of CH_4_ by generating intra-oxic conditions in the cell from nitrite (Ettwig et al. 2010, Shen et al. 2016, Guerrero-Cruz et al. 2021). Furthermore, AOM is performed by some archaeal taxa named anaerobic CH_4_ oxidizers (ANME). The most well-known ANME member is *Candidatus* Methanoperedens nitroreducens (formerly ANME-2d), an anaerobic archaeon that utilizes nitrate to oxidize CH_4_ via reverse methanogenesis (Haroon et al. 2013). Additionally, some methanogens have even been indicated to be able to switch between methanogenesis and AOM (Kevorkian et al. 2022).

The abundance of these CH_4_-cycling microorganisms and their interplay with environmental parameters and other microbes is thus crucial to understanding CH_4_ emissions of a specific environment, such as KHs in a complex terrestrial landscape (Reverey et al. 2016). To unravel the potential impact of land use on CH_4_ production in KHs, the main drivers and the respective CH_4_-cycling microbiota, we sampled sediments of 10 water-filled KHs in two different surrounding land use types (forest and agriculture), determined their potential net CH_4_ production rates and the composition of their microbiota, with a focus on the functional groups that perform CH_4_ formation and oxidation. We hypothesize that environmental conditions in agricultural KH sediments, in particular higher inorganic solute load, may lead to higher potential CH_4_ net production and promote different CH_4_-cycling microorganisms including anaerobic CH_4_ oxidizers in comparison to forest KHs.

## Materials and methods

### Study site

The 10 investigated KHs (Table 1; all water-filled) are located within the river Quillow catchment (168 km^2^), which is part of the hilly, young moraine landscapes typical for Northeastern Germany. The climate is subhumid (mean annual temperature: 8.6°C; mean annual precipitation ranges from 350 to 750 mm yr^−1^) (Frindte et al. 2023). KHs are abundant and make up ~1.4% of the entire study area (Nitzsche et al. 2016) and form important components of a complex landscape (Premke et al. 2016). A detailed description of the area and its history can be found in Kleeberg et al. (2016).

**Table 1.** Sampled water-filled kettle holes (KHs).

Name	Coordinates		Area [ha]	Land use	Vegetation/crop	Sample IDs
**for1**	53°16′22.04″N	13°28′56.59″E	0.16	forest	mixed forest	1–4
**for2**	53°16′17.45″N	13°28′46.46″E	0.18	forest	mixed forest	5–8
**for3**	53°16′16.68″N	13°28′40.44″E	0.13	forest	mixed forest	9–11
**for4**	53°16′10.20″N	13°28′44.40″E	0.06	forest	mixed forest	13–16
**for5**	53°16′5.53″N	13°28′46.21″E	0.51	forest	mixed forest	17–20
**agr1**	53°23′40.79″N	13°39′43.26″E	0.32	agriculture	rapeseed	21–24
**agr2**	53°23′12.24″N	13°41′10.74″E	0.72	agriculture	barley	25–28
**agr3**	53°22′41.50″N	13°42′16.17″E	0.36	agriculture	rapeseed	29–32
**agr4**	53°23′50.56″N	13°39′56.87″E	0.15	agriculture	rapeseed	33–36
**agr5**	53°24′22.88″N	13°39′4.84″E	0.38	agriculture	rapeseed	37–40

### Sampling

Sampling took place on 24 and 31 July 2017. Undisturbed sediment cores were taken in quadruplicate from the inundated pond center (water column of 30–50 cm) of each KH using a gravity corer (Uwitec GmbH) equipped with a PE tube (∅ 7 cm). For one KH (for3), only three cores could successfully be sampled. For each core, the first 10 cm was sliced off and homogenized. For gas measurements in the lab, parts of the homogenized sediment were transferred into PE incubation tubes (∅ 5.4 cm) and covered with KH surface water, creating a sediment column of 10 cm, a water column of 1 cm and a headspace of 14 cm. The residual sediment was subsequently stored on ice for transport to the lab for physicochemical analyses and determination of organic matter properties. For molecular biological analysis, *Ca*. 0.3 g of the homogenized sediment was filled into 2 ml Eppendorf tubes, stored on dry ice for transport, and subsequently frozen at -80°C.

### Gas flux measurements

For acclimatization, the incubation cores were closed with a gas-tight lid and stored in a climate chamber (20°C) in the dark for 3 days prior to measurements. The lid was equipped with an in- and outlet for connecting an Ultraportable Greenhouse gas analyzer (UGGA; Los Gatos Research Inc.), thus creating a closed loop including the headspace in the incubation tube. CO_2_ and CH_4_ concentrations in the headspace (ppm) were measured continuously for 3 min for each sample. The increase was fitted via linear regression (all R^2^ > 0.8). The potential net CH_4_ and CO_2_ production rate from the sediment Net_gas_ in mg C m^−2^ d^−1^ was calculated according to equation 1:


(1) \begin{eqnarray*}
Ne{t_{gas}} = {10^{ - 6}}*\frac{{s*{V_{HS}}}}{{{V_{m0}}*A}},
\end{eqnarray*}

where s is the gas production (ppm d^−1^), V_HS_ is the volume of the headspace (ml), V_m0_ is the molar volume of an ideal gas (l mol^−1^) and A is the area of the incubation tube (m^2^).

### Sediment texture and chemical parameters analyses

The main textural fractions (sand, silt, and clay) were determined via laser diffraction with wet dispersion using a Mastersizer 3000 laser particle size analyzer (Malvern Instruments Ltd.) according to ISO 11277. Water content was determined by subtracting the weight of the freeze-dried sediment from its wet weight. Organic matter was determined via loss on ignition (550°C for 4 h). Mineral nitrogen was extracted with potassium chloride (KCl), and nitrate- and ammonium-bound N were measured with a CFA-SAN spectrophotometer (Skalaranalytic GmbH). Sulfate-bound S was extracted with KCl and measured with a Dionex ICS-2100 ion chromatography system (Thermo Fisher Scientific Inc.). The pH value was determined by water extraction according to ISO 10390. For the following analyses, the sample was air-dried, sieved (2 mm), and ground. Total carbon (TC) and nitrogen (TN) were measured with a TruSpec elementary analyzer (Leco Instruments GmbH). Total organic carbon (TOC) was determined via fractionated simultaneous determination with an RC612 multiphase carbon and water analyzer (Leco Instruments GmbH). Total inorganic carbon (TIC) was calculated from the difference of TC and TOC.

### Determination of organic matter properties

Water extractable organic matter (WEOM) represents the mobile and biologically available fraction of total sediment OM. The extraction and measurements were performed as described in Reverey et al. (2018). Briefly, fluorescence excitation-emission matrices were measured (Aqualog spectrometer, Horiba GmbH) with an emission range from 210 to 620 nm (1.6 nm increments) and an excitation range from 250 to 600 nm (5 nm increments). Blanks were measured with Milli-Q Millipore water. Spectral correction, inner filter correction, Raman normalization, and blank subtraction of the EEMs were automatically conducted by the instrument software. For estimation of WEOM characteristics, three indices and specific UV absorbance at 254 nm were calculated (Table 2).

**Table 2.** Calculated water extractable organic matter (WEOM) indices and their characteristics.

Index	Calculation	Corresponds to	Range	References
**Fluorescence index (FIX)**	ratio of the emission intensities at 470/520 nm, excitation wavelength of 370 nm	Source of DOM	∼1.2–∼1.8 (low values indicate plant/soil OM as source, high ones microbial sources)	Fellman et al. (2010), Brandl et al. (2020)
**Humification index (HIX)**	the ratio between the two spectral areas between emission intensities of 435–480 nm and 300–345 nm at an excitation wavelength of 254 nm	Degree of humification	High values indicate high humification	Ohno (2002), Fellman et al. (2010)
**Biological index (BIX)**	ratio of the emission intensities at 380/430 nm, excitation wavelength of 310 nm	proportion of recently produced DOM	0–∼1 (values >1 are considered high and indicate recent production)	Brandl et al. (2020), Hansen et al. (2016)
**Specific UV Absorbance at 254 nm (SUVA)**	Ratio of absorbance at 254 nm to DOC * cuvette path length [m]	degree of aromaticity of the dissolved OM	1–9 l mg^−1^ m^−1^ (high values indicate high aromaticity)	Weishaar et al. (2003), Hansen et al. (2016)

### DNA extraction from sediment samples and sequence data analysis

Genomic DNA was extracted using a CTAB-phenol-chloroform-isoamyl alcohol/bead-beating protocol [modified after Nercessian et al. (2005)] as described in Reverey et al. (2018). PCR, library preparation, and sequencing were performed by LGC Genomics (Berlin, Germany). Briefly, the V3–V4 region of the 16S rRNA gene was amplified for Bacteria using primers 341F-785R (Klindworth et al. 2013) and for Archaea using a semi-nested approach with primers 340F-1000R (Gantner et al. 2011) and primers U341-U806R (Sundberg et al. 2013), followed by library preparation and sequencing (2 × 300 bp) on a MiSeq platform (Illumina). Demultiplexed raw sequence data was quality checked and analyzed using the DADA2 package (Callahan et al. 2016) in R using the following parameters: for Bacteria truncLen = c(260 230), maxN = 0, maxEE = c(3,3), and truncQ = 2 and for Archaea truncLen = c(260 230), maxN = 0, maxEE = c(2,2), and truncQ = 2, generating sequences of ~405 and 387 nt, respectively. Taxonomic assignment of the generated amplicon sequence variants (ASVs) was done using the SILVA Online classifier (https://www.arb-silva.de) with the SSU database v138.1 (Quast et al. 2013). ASVs assigned as mitochondria, chloroplasts, or unclassified were subsequently removed from the data table. Furthermore, bacterial ASVs were removed from the archaeal data table, while archaeal ASVs were removed from the Bacteria data table. The sequence data were deposited in GenBank under the BioProject number PRJNA1187232.

Methanotrophs and methanogens ASVs were separated into count tables using lists of known methane-cycling taxa. The reference taxa lists can be found in tables A1 and A2. Methanogen taxa classified as “Rice Cluster I” were renamed as “*Methanocella*” according to Sakai et al. (2008). This concerned three ASVs. Relative abundances of methanogens, methanotrophs, and anaerobic methanotrophs were determined by calculating their proportion of total 16S rRNA ASV counts of bacteria or archaea, respectively.

### Statistical analyses

Statistical analyses were performed in R version 4.3.0 (R Core Team 2023). Normal distribution of data was checked using the Shapiro–Wilk test. The zero hypothesis of normal distribution had to be rejected for all variables. Consequently, significant differences in variables by land use type were determined using a Wilcoxon test. Testing for significant differences of variables between single KHs was performed using a Kruskal–Wallis test followed by a post-hoc Dunn's test (with Bonferroni *P*-value adjustments for multiple group comparisons). To find patterns in the dataset, a principal component analysis (PCA) based on the correlation matrix of the soil and microbial community variables was performed. The analysis of the sequencing data and calculation of alpha-diversity indices were performed using the R packages “phyloseq” V1.46.0 (McMurdie and Holmes 2013) and “vegan” V2.6.4 (Oksanen et al. 2018).

From samples that showed saturated rarefaction curves, the one with the lowest total read counts was identified (2859 for bacteria and 5058 for archaea), and 90% of this count was set as sample size for sample rarefaction. Samples with unsaturated rarefaction curves were excluded from all analyses. Shannon alpha-diversity indices were calculated from the rarefied samples. For the microbiota composition analyses, global singletons and doubletons were removed from the ASV tables. KHs with only one sample left were excluded from analyses, as one sample was not considered to sufficiently represent potential heterogeneities in KHs. To access differences in microbial composition of the samples, non-metric multidimensional scaling (NMDS) plots were drawn using Bray–Curtis distance based on relative abundances. Impact of categorical variables (land use, single KHs) on the microbiota was analyzed by permutational analysis of variance (PERMANOVA), using the adonis2 function of the “vegan” package (999 permutations, Bray–Curtis distance matrix). Significant differences in abundance of specific genera were tested using the Welch test with the R package “microbiomeMarker” V1.8.0 (Cao et al. 2022) and Bonferroni *P*-value adjustments for comparing multiple groups. The *P*-value cutoff was set to 0.05.

The impact of physicochemical variables on the CH_4_-cycling microbiota was assessed by redundancy analysis (RDA) in R. Missing data were extrapolated by averages of samples of the same land use, in order to avoid loss of samples due to missing values. This was done for three samples of forest land use type: sample no. 4 on the methanogen relative abundance and sample no. 1 on archaeal Shannon index, as well as sample no. 14 on bacterial Shannon index and relative methanotroph abundance. The numbers correspond to the sample numbers in Table 1 and belong to KHs for1 (no. 1 and 4) and for4 (no. 14). The environmental data were standardized, and ASV table counts were combined on the genus level followed by transformation (logarithmic and Hellinger). Genera that were only present in one sample were removed. No forward selection was performed, instead variables were manually selected, excluding variables that showed strong covariances with others. Variables influencing the model significantly were identified by ANOVA.

To identify major drivers of potential net CH_4_ production, partial least square regression (PLSR) was conducted using the “plsr()” function of the R package “pls” V2.8.3 (Liland et al. 2023). For a description of the method, see Attermeyer et al. (2017). In short, PLSR is a method well suited for a dataset with comparatively few observations, but a high number of variables, which may be collinear. Prior to analysis, variables with a skewness greater than 1 were log(x) or log(x+1) transformed, which concerned CH_4_ net production (response variable), BIX, TIC, ammonium, sulfate, methanogen, and methanotroph relative abundance. All predictors were scaled and centered. Variable importance on projections (VIP) was calculated using the “VIP()” function of the “plsVarSel” package V0.9.11 (Mehmood et al. 2012). Values exceeding 1 were considered highly influential, between 1 and 0.8 as moderately influential, and <0.8 as less influential.

## Results

### Potential net CH_4_ and CO_2_ production and physicochemical parameters

**Figure 1.**
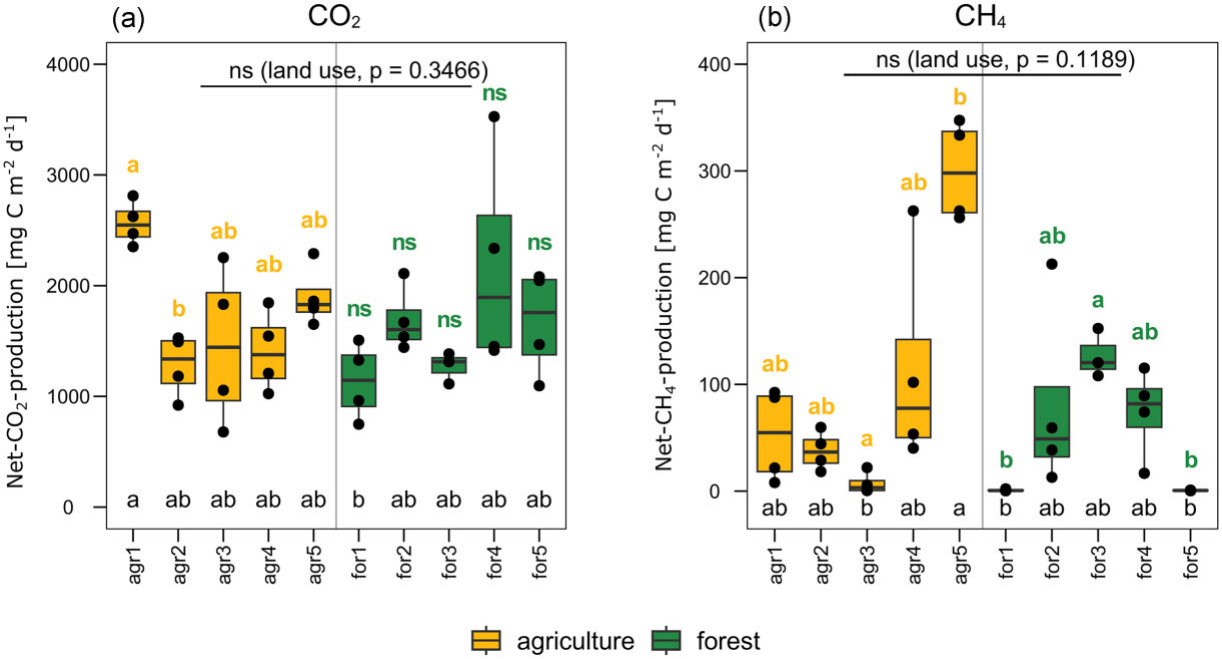
Potential net production rates of CO_2_ (a) and CH_4_ (b) in kettle hole (KH) sediment samples during incubation. KHs from different land use surroundings are indicated by color of the boxplots. Significant differences between land use groups (Kruskal–Wallis test) are indicated on top of the plot with *P*-value. Significant differences between all single KHs are indicated along the *x*-axis, and significant differences between KHs within the same land use group are indicated in the respective color above the boxes. Significant differences (*P* < .05) were detected between samples labeled with contrasting letter labels on top of the boxes (“a” or “b”). Non-significant differences are indicated with “ns”.

The potential net CH_4_ production measured in the incubated samples ranged from 0.28 to 212.72 mg C m^−2^ d^−1^ in forest (for) and 0.57 to 347.39 mg C m^−2^ d^−1^ in agricultural (agr) KHs, respectively (Fig. 1). There was no significant difference detected according to land use, but differences between single KHs were apparent and in some cases significant. The highest rates were detected in KH agr5, while the lowest CH_4_ production rates were obtained from KHs for1, for5, and agr3. Statistically significant differences were detected between agr3 and agr5 (both *P* < .05) and between agr5 and for1 as well as between agr5 and for5 (all *P* < .01). The KHs agr4 and for2 did show considerable differences between their replicates.

The total range of potential net CO_2_ production covered 748.83–3526.89 mg C m^−2^ d^−1^ for KHs with forest and 678.98–2811.66 mg C m^−2^ d^−1^ with agricultural surroundings. No statistically significant difference was detected by land use type. Significant differences were detected between KHs agr1 and for1 (*P* < .05). KH agr1 had the highest and KH for1 the lowest mean CO_2_ production (Fig. 1a). Potential net CO_2_ production was generally ~5- to 30-fold higher than potential net CH_4_ production in all samples (Fig. 1).

All measured inorganic solute contents (ammonium, nitrate, sulfate) were significantly higher in KHs surrounded by agriculture (Table 3). Nitrate was highest concentrated in agr3 (Table S3). Ammonium concentrations were notably higher than nitrate concentrations. The highest concentrations were detected in KH agr5, highly exceeding the second-highest concentrations in KH agr2. Sulfate concentrations were low in all forest KH samples, while samples of agricultural KHs showed a large variation, sometimes even within single KHs (Fig. S1). Matching the nitrate and ammonium concentrations, total nitrogen was significantly increased in agricultural KHs, while total C and total organic C were higher in agricultural KHs, although not significantly. Soil texture parameters clay and silt were significantly higher in agricultural KHs, while sand content was significantly higher in forest KHs. These variables did not show much variation in KHs of the same land use area. Statistical testing did not reveal significant *P*-values for tests of single KHs within the same land use, except clay and sand in agricultural KHs. Similarly, all WEOM properties were significantly different at land use level, while only HIX was significantly different between individual forest KHs.

**Table 3.** Means of physicochemical parameters according to land use.

	Parameters			Means ± SD (land use)	
	Abbreviation		Unit	Forest	Agriculture
inorganic solutes		ammonium	mg 100 g^−1^ _soil wet weight_	***6.22 ± 3.73***	***17.01 ± 15.08***
		nitrate	mg 100 g^−1^ _soil wet weight_	***0.04 ± 0.02***	***0.12 ± 0.05***
		sulfate	mg 100 g^−1^ _soil wet weight_	***0.26 ± 0.29***	***22.36 ± 39.75***
soil variables		pH		**6.20 ± 0.34**	***7.13 ± 0.49***
	TN	Total nitrogen	%	***0.55 ± 0.21***	***1.12 ± 0.41***
	TIC	Total inorganic carbon	%	***0.10 ± 0.05***	***0.57 ± 0.60***
	TOC	Total organic carbon	%	*9.07 ± 3.92*	*11.25 ± 4.16*
	TC	Total carbon	%	*9.17 ± 3.97*	*11.82 ± 4.37*
	WC	Water content	%	***62.26 ± 12.55***	***76.02 ± 10.04***
	OM	Organic matter	%	***18.40 ± 7.61***	**23.98 ± 8.37**
gas fluxes	CO_2_	Carbon dioxide	mg C m^−2^ d^−1^	1607.21 ± 601.96	1721.13 ± 589.84
	CH_4_	Methane	mg C m^−2^ d^−1^	52.92 ± 62.26	102.38 ± 115.03
soil texture		Clay	% volume	**5.44 ± 1.05**	***12.04 ± 4.23***
		Silt	% volume	**51.88 ± 6.94**	**68.73 ± 9.75**
		Sand	% volume	**42.69 ± 7.38**	***19.21 ± 12.41***
WEOM properties	FIX	Flourescence index		**1.45 ± 0.04**	**1.56 ± 0.08**
	BIX	Biological index		**0.47 ± 0.03**	**0.68 ± 0.11**
	HIX	Humification index		***6.28 ± 1.65***	**3.72 ± 1.51**
	SUVA	Specific UV absorbance		**3.70 ± 0.38**	**2.18 ± 0.50**

*Note*: Significant differences between land use type are indicated by bold letters (Wilcoxon test *P* < .05), while statistically significant differences between single kettle holes of the same land use group are indicated with italic letters (Kruskal–Wallis test, *P* < .05).

**Figure 2.**
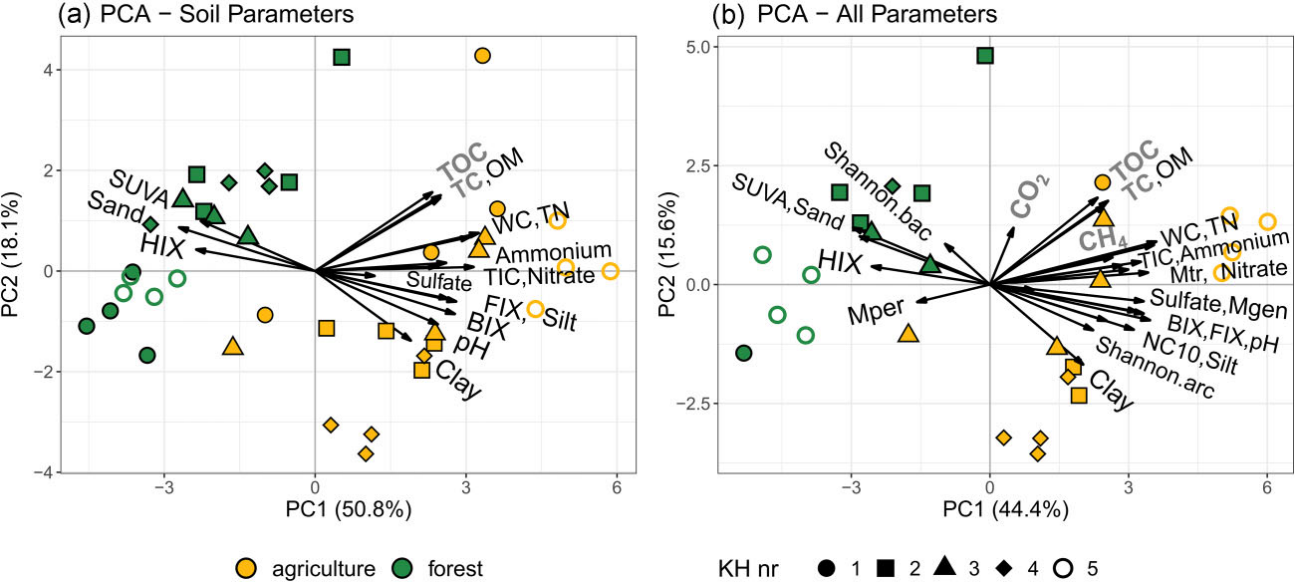
Biplot of the principal component analysis (PCA) of soil physicochemical variables (a) and all variables including microbial and gas flux parameters (b) of sediments of 10 kettle holes (KH) in two land use areas. For B, only samples with successful sequencing efforts could be considered. Land use groups are indicated by color, single KHs by symbol shape. Parameters that were not statistically different between land use types (Wilcoxon-test, *P* < .05) are indicated in gray, bold letters. Mgen, Methanogen relative abundance; Mtr, aerobic methanotroph relative abundance; NC10, *Methylomirabilaceae* relative abundance; Mper, *Ca*. Methanoperedens relative abundance; Shannon, alpha-diversity index for Bacteria (.bac) and Archaea (.arc). Soil parameter abbreviations can be found in Table 3.

The PCA of the soil variables showed a clear clustering of samples by land use type (Fig. 2a), mostly along the first axis which explained the majority of variance (50.8%). The samples within land use varied more on the second axis (explaining 18.1% of the observed variance). The drivers of separation were sand, HIX, and SUVA for the forest samples while the agricultural samples were mainly influenced by clay, pH, BIX, and FIX. TC, TOC, and OM did not play a role in sample separation by land use type, but by separating the samples within each land use group. Several parameters were strongly intercorrelated, including TOC, TC and OM, TN and WC, ammonium, nitrate, and TIC as well as sand, SUVA, and HIX.

### Relative abundances of the methane-cycling microbiota and microbial alpha-diversity

**Figure 3.**
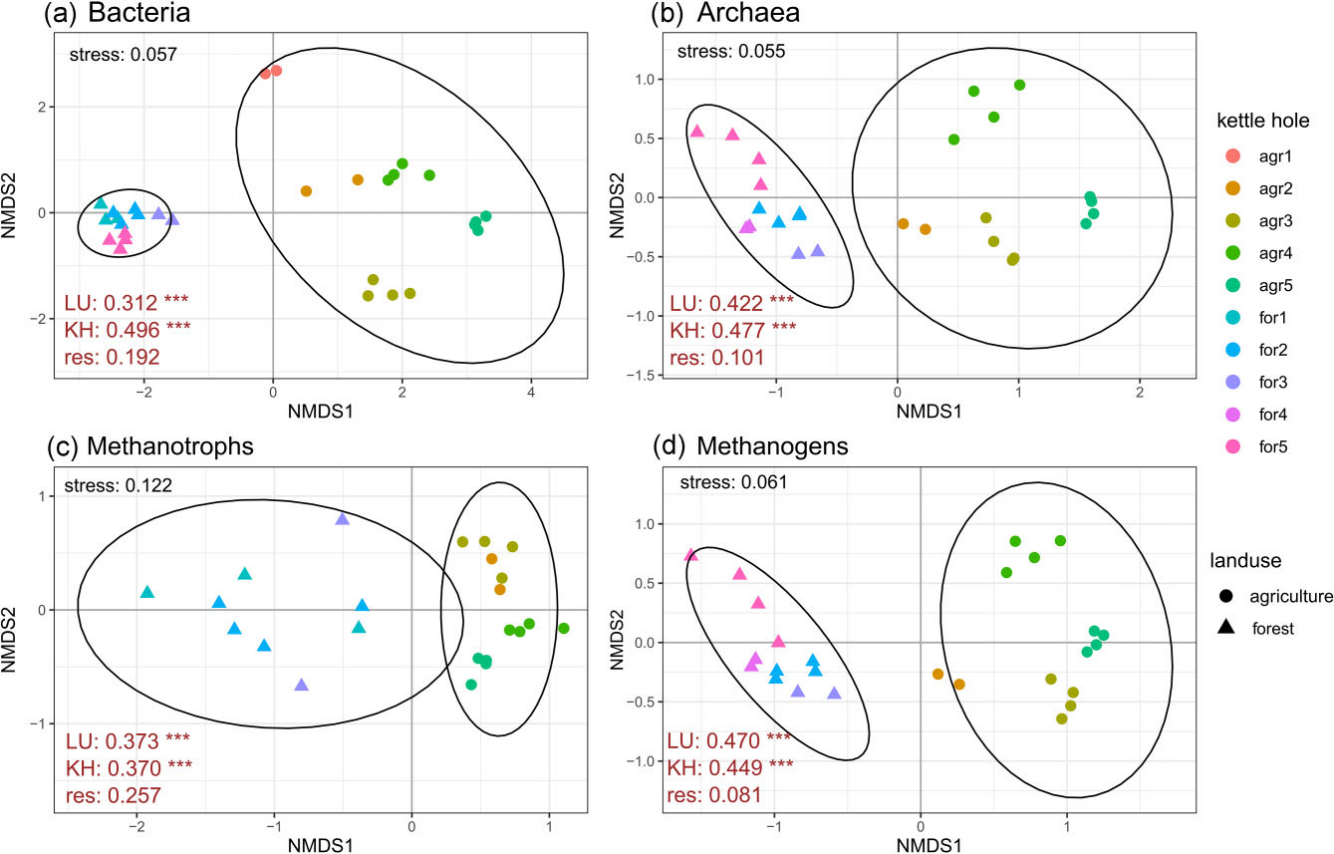
NMDS plots visualizing the microbiota composition for Bacteria (a), Archaea (b), methanotrophs (c), and methanogens (d) in samples from kettle holes of different land use (shape of symbols). Samples from the same kettle hole are indicated by color of symbols. Ellipses indicate 95% confidence intervals for samples of the same land use group. PERMANOVA results (*R*^2^) based on Bray–Curtis distance for the respective datasets are listed in red letters on the lower left of the plots (LU, land use; KH, kettle hole; and res, residuals). Respective stress values are listed on the top left of the plot. Only samples with successful sequencing efforts and more than one usable replicate per KH are included. For the methanotrophs (c), samples with no or very low (<10) counts were not considered.

The relative abundances of aerobic methanotrophs, *Methylomirabilaceae*, and methanogens were significantly higher in agriculture KHs (Fig. 3b and Fig. S2). The relative abundance of aerobic methanotrophs reached 0%–2.25% in samples from forest KHs (with no methanotroph ASVs in two samples of KH for5) and 0.02%–8.53% in agriculture KHs. Mean aerobic methanotroph relative abundance was the highest in KH agr5 (7.38%). The abundance of methanotroph of KH agr5 was significantly higher than in all other agricultural KHs (0.02%–2.01%). In forest samples, KH for3 had the highest methanotrophs relative abundance (mean of 1.51%) which was significantly higher than in KH for1 (0.01%).

Methanogen ASVs were present in all samples and made up ~50% of total archaeal 16S rRNA ASVs. Differences in methanogen relative abundances were only significant between the forest KHs for3 and for5. The range was generally larger for forest KH samples (19.39%–64.03%) compared to agricultural KHs (54.76%–75.73%). ASVs of the anaerobic CH_4_ oxidizer *Ca*. Methanoperedens was only detected in some forest KHs (KHs for1, for2, and for5), with the biggest range for KH for1 of 0%–7.69%. In the further samples, the highest relative abundance was 1.01% (for5), the others ranged between 0.02% and 0.47%.

The Shannon alpha-diversity index for Bacteria ASVs was significantly higher in forest KH samples (Fig. S3). No significant differences between KHs within land use types were detected. For Archaea, the Shannon indices were generally lower, but significantly increased in agricultural KHs. Significant differences were only found between agr3 and agr5; none between forest KHs.

The PCA which included microbial ASV and potential net greenhouse gas (GHG) production data revealed overall a clear clustering of forest and agricultural KH samples (Fig. 2b), similar to the ordination only by soil variables (Fig. 2a). The first axis (explaining 44.4%) is mostly associated with methanotroph and methanogen relative abundance in addition to the soil parameters already mentioned (for Fig. 2a). The second axis (explaining 15.6%) is most directly associated with potential net CO_2_ production. The arrows indicated a positive correlation of methanotroph relative abundance with nitrate, ammonium, and TIC as well as total nitrogen, water content, and potential net CH_4_ production. For methanogen relative abundance, a correlation with BIX, FIX, and pH is indicated, as well as with nitrate and methanotroph relative abundance. *Methylomirabilaceae* relative abundance is positively correlated with silt and to a lesser degree with BIX, FIX, pH, and methanogen relative abundance as well as Shannon index for Archaea. A negative correlation to potential net CH_4_ production was indicated for the relative abundance of *Ca*. Methanoperedens, as well as to the relative abundance of aerobic methanotrophs.

### Composition of the methane-cycling microbiota

After quality control, several samples had to be removed, and KHs with only one replicate left were excluded from further analysis. This resulted in the removal of two KHs from the archaeal and methanogen datasets (for1 and agr1) and one from the bacterial and methanogen datasets (for4). In case of the methanotrophs, some samples also had to be removed because of very low total ASV counts per sample, i.e. <10. For KH samples 18 and 20 (both from KH for5), no ASV that affiliated to methanotrophs was identified and thus, KH for5 and agr1 datasets were also excluded. The number of observed ASVs was notably lower for methanotrophs (0–16) than for methanogens (26–83) (Fig. S5).

NMDS plotting revealed distinct clusters reflecting the land use type (Fig. 3) for Bacteria and Archaea as well as methanotrophic and methanogenic taxa. In all four plots, there is a clear separation of the land use type along the first axis. With the exception of the methanotrophs, the samples from forest KHs showed more distinct clusters for all microbial groups, whereas it was opposite for agricultural KHs. In all plots, the replicate samples of single KHs, especially from agricultural land use type form distinct clusters, which is less pronounced for the methanotroph dataset. The plots for Archaea and methanogens shared a highly similar pattern. The influence of land use was confirmed for all datasets with PERMANOVA, where land use type was highly significant (*P* = .001) and explained a large portion of variance in the samples. Individual KHs had similar *R*^2^ values and *P*-values. In case of methanogens and methanotrophs, land use explained even more variance than individual KH, although especially for methanotrophs, the unbalanced sample distribution has to be noted.

The detected methanotroph ASVs were assigned to seven different genera of the families *Methylococcaceae* (genera *Methylocaldum* and *Methyloparacoccus*) and *Methylomonadaceae* (*Crenothrix, Methylobacter*, and *Methylomonas*) of the class *Gammaproteobacteria* (Fig. S2). Some were not assigned on genus level, but belonged to the *Methylomonadaceae*. No alphaproteobacterial methanotrophs were detected. In addition, members of the family *Methylomirabilacaea*, which includes the anaerobic methanotroph *Ca*. Methylomirabilis oxyfera, were detected (groups Sh765B-TzT-35 and Z114MB74, with the latter in only one sample). Group Sh765B-TzT-35 constituted 37.1% and 39.4% of methanotroph ASV counts in KHs agr2 and agr3, while it was not occurring in some individual forest samples. Group Sh765B-TzT-35 was significantly more abundant in agricultural KHs than in forest ones (*P* < .05). The most methanotrophic genera occurred in samples from both land use types, except for *Methylocaldum* and *Methyloparacoccus*. The latter were only detected in few agricultural samples with low relative abundances (<1% of methanotrophic ASVs). The respective abundance of other genera varied considerably. Most notably, the relative abundance of *Crenothrix* was significantly higher (*P* < .01) in samples from agricultural KHs. In those samples, it was often the dominant methanotroph. On the other hand, *Methylobacter* abundance was significantly higher in forest KH samples compared to the agricultural ones (*P* < .01). Thus, in forest KH samples, *Methylobacter* and *Crenothrix* were the most abundant genera, while in agricultural KHs, it was *Crenothrix* and group Sh765B-TzT-35.

Thirteen genera of methanogens were detected, in addition to unclassified genera. They belonged to eleven different families and are mostly hydrogenotrophic (e.g. genera *Methanobacterium, Methanocella*, and *Methanolinea*). Some aceticlastic (*Methanosaeta*) and methylotrophic genera (*Methanomassiliicoccus, Ca*. Methanofastidiosum, and *Ca*. Methanomethylicus) were also detected, in addition to the metabolically versatile *Methanosarcina*. The most abundant genera across all samples were *Methanoregula* (mean of 31.2%) and *Methanosaeta* (25.0%). The latter was significantly more abundant (*P* < .01) in agricultural samples. Notably, in KH for5, their abundances were <20%, and *Methanosarcina* abundances were increased (29.6%) compared to other KHs. KH for5 samples included also the highest relative abundances of *Methanocella* (13.9%), *Methanobacterium* (7.2%), and *Methanomassiliicoccus* (7.0%). The genus *Ca*. Methanofastidiosum was only detected in agricultural samples (agriculture mean: 0.6%) and was significantly more abundant there (*P* < .05). *Methanolinea* was significantly more abundant in agricultural KH samples, where it was the third most abundant methanogen (7.6%). *Methanobacterium* was significantly more abundant in forest KHs (*P* > .01). The relative abundance of *Methanocella* was also notably increased in forest KHs, but this increase was not significant.

### Impact of environmental variables on methane-cycling microbiota

The relationship between the composition of the CH_4_-cycling microbiota and environmental variables was explored by RDA. Excluded variables were HIX, BIX, and FIX due to their strong covariance with SUVA, TC, and TOC due to their covariance with OM, clay, and silt due to their covariance with sand and TN due to its covariance with ammonium. The model explained 80.4% of variance in the methanotroph ASV abundances (48.7% by the first axis and 19% by the second axis) and 81.7% of the methanogen ASV abundances (45.9% by the first axis, 16.1% by the second). Overall significance was confirmed by ANOVA for both models (*P* = .002 for the methanotroph model and *P* = .001 for the methanogen model). The RDA biplots (Fig. 5) revealed that for both microbial groups, methanotrophs and methanogens, samples clustered largely according to land use type. The main parameters separating the clustering by land use type were sand and SUVA.

**Figure 4.**
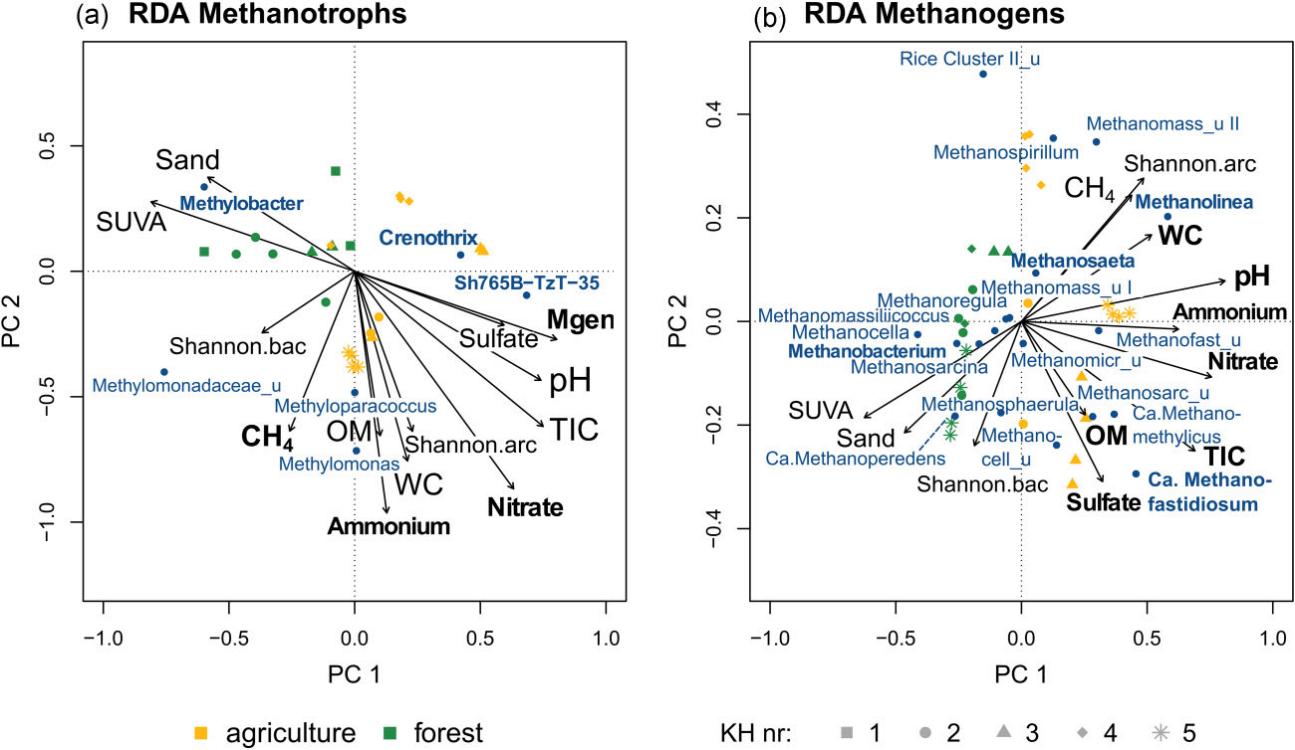
Redundancy analysis (RDA) biplots of methanotroph (a) and methanogen (b) genera based on selected environmental variables. The samples are distinguished by land use (color) and single KH (symbol shape). Variables with a significant impact in explaining the variance in the model and genera that were detected significantly more abundant in a particular land use type are indicated in bold letters. Blue, genus names; uncultured genera are indicated with “_u” after an abbreviation of their respective taxon. Genera present in no more than one sample were removed. Explanatory variables are indicated by black arrows and labels; abbreviations for soil variables can be found in Table 3. Mgen, relative abundance of methanogens; Shannon, alpha diversity index of Bacteria (.bac) and Archaea (.arc). Methanomass, *Methanomassiliicoccaceae*; Methanocell, *Methanocellaceae*; Methanofast, *Methanofastidiosales*; Methanomicr, *Methanomicrobiales*, Methanosarc, and *Methanosarcinaceae*.

Variables with significant effects on methanotroph ASVs were ammonium and nitrate concentrations as well as the methanogens’ relative abundance (all *P* < .01) and potential net CH_4_ production (*P* < .05). None of the explanatory variable was strongly associated with the first axis, reflected by the strongest canonical coefficient for the axis being -0.56 for SUVA. The second axis, however, was strongly associated with ammonium, nitrate, and WC (all canonical coefficients >0.65). The methanotroph genera that were mainly associated with the agricultural KH samples were *Crenothrix, Methyloparacoccus*, and *Methylomonas. Methylobacter* was located notably among forest samples, associated most closely with the variables sand and SUVA (Fig. 4a). *Crenothrix* and group Sh765B-TzT-35 abundance was not directly driven by a specific explanatory variable, but were associated with high pH, TIC, sulfate, and methanogen abundance. *Methylomonas* and *Methyloparacoccus* were associated with organic matter content, water content, ammonium concentration, and potential net CH_4_ production.

For the methanogen model ammonium, nitrate, sulfate and pH (all *P* < .01) as well as TIC, organic matter and water content were significant explanatory variables (all *P* < .05; Fig. 4b). The sample clustering according to land use type is most apparent on the first axis, which is strongly associated with ammonium, nitrate, pH, TIC and SUVA (all with canonical coefficients stronger than ±0.7), while the general associations to the second axis were less strong (e.g. strongest coefficient -0.46 for sulfate). The genera that clustered especially among forest samples and therefore are associated most with sand and SUVA include *Ca*. Methanoperedens, *Methanobacterium, Methanocella* and *Methanosarcina. Methanolinea, Ca*. Methanomethylicus, *Ca*. Methanofastidiosum, and *Methanospirillum* were located among agricultural samples, the latter ones notably among agr4 samples. *Methanosphaerula* was located in between agricultural and forest samples and was associated with high bacterial Shannon index. The strictly acetoclastic methanogens belonging to *Methanosaeta* were closely associated with high potential of net CH_4_ production and archaeal Shannon diversity. *Methanomassiliicoccus, Methanosarcina, Methanoregula*, and several uncultured genera seemed to be less impacted by the explanatory variables as they were located more central in the ordination.

### Variables impacting potential net CH_4_ production rates

**Figure 5.**
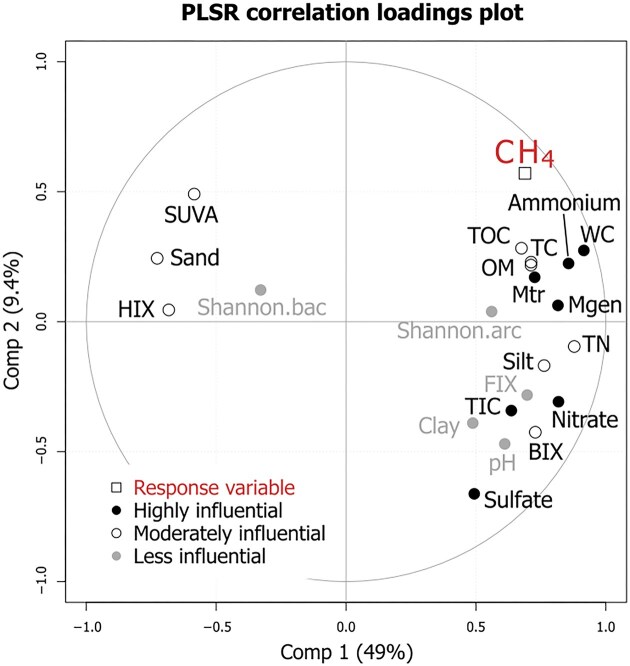
Correlation loadings plot of partial least squares regression (PLSR). Two components were used. The response variable was potential net CH_4_ production, explanatory variables were physicochemical and microbial parameters. Variable importance on projection (VIP) scores are indicated by symbol shape and color. Values >1 were considered highly influential, >0.8 as moderately and <0.8 as less influential. The abbreviations used for environmental variables can be found in Table 3. Mtr, relative abundance of aerobic methanotrophs; Mgen, relative abundances of methanogens.

PLSR revealed the contributions of each tested variable to the prediction model of net CH_4_ production (Fig. 5). Variables that were considered highly influential were ammonium and sulfate (with VIP scores of 1.33 and 1.66, respectively), the relative abundances of methanogens and aerobic methanotrophs as well as water content, nitrate and TIC. Less influential variables were bacterial and archaeal Shannon index, clay, FIX, and pH. The correlation loadings plot (Fig. 5) revealed that the most explanatory variables have high correlation loadings (up to 0.92 for water content) on the first component, which were negative for sand, SUVA, HIX, and Shannon index for Bacteria and positive for the other variables. The correlation loadings on the second component were generally lower, the strongest being -0.66 for sulfate. The predictor variable was positively correlated with both components, especially strong to the first. The location of predictor variables in the biplot indicates correlation with the explanatory variable; here, the variables closest to CH_4_ are related to carbon or organic matter content, ammonium, and water content as well as relative abundance of aerobic methanotrophs and methanogens, indicating a positive correlation.

## Discussion

This study represents a step forward in understanding the variability of KHs for aquatic CH_4_ budgets in regard to the role of land use type in the surrounding landscape. Our study highlights substantial differences on land use level based on sediment and microbial parameters in KHs (Figs 2 and 3). However, the differences of the measured potential net CH_4_ production suggested that these were controlled on a more individual level in KHs, whereas land use had a clear effect on the CH_4_-cycling microbiota composition and biodiversity.

### Abundance of methane-cycling microbiota

The high range in relative abundances of methanotrophs (both aerobic and anaerobic) in our study indicates that methanotrophy is a process of variable importance in the inundated zone of KHs. We detected no or comparatively few methanotrophs in KHs sediment cores that were characterized by a very low potential net CH_4_ production, most notably KH for5. As methanotrophy and by extension relative abundance of methanotrophs are dependent on CH_4_ availability (Hanson and Hanson 1996), substrate limitation can be assumed in these KHs. In parallel, KH agr5 featured the highest potential net CH_4_ production and was characterized by high relative abundances of aerobic methanotrophs. This is in line with the correlation of methanotroph relative abundance and the potential net CH_4_ production rates (Fig. 2b) and indicates that the low potential CH_4_ rates in some KHs might be connected to low methanogen activity rather than high CH_4_ consumption by aerobic methanotrophs. This conclusion is further corroborated by the correlation of aerobic methanotroph abundance and ammonium concentration, which is a competitive inhibitor of methanotrophy (Semrau et al. 2010).

Methanogen abundance was not increased along with potential net CH_4_ production in KH agr5 compared to other agricultural KHs, and while it was lower in low-emitting KHs, there was still a considerable number of methanogen ASVs detected. The reason might further be related to organic C content, as KHs for1 and for5 showed the lowest TOC, TC, and OM levels of all sampled KHs. This may have led to reduced microbial degradation of labile OM and thus lower organic substrate availability for methanogenesis. Additionally, the correlation between methanogen relative abundance with BIX and FIX (Fig. 2b) indicates a relationship with organic matter quality. Soil texture and WEOM properties in for1 and for5 were not different from other forest KHs with higher CH_4_ production and methanogen abundance, but water content was notably lowest in these KHs. This indicates a denser sediment which potentially limits access to inorganic solutes and labile OM. Corroborating this conclusion, ammonium and total nitrogen levels were also lowest in these samples.

Notably, in the forest samples with low CH_4_ production, *Ca*. Methanoperedens was detected, indicating a high importance of anaerobic methanotrophy in these samples as abundance of aerobic methanotrophs was comparatively low. The negative relationship to CH_4_ production potential and aerobic methanotrophs (Fig. 2b) indicates that they are outcompeted by aerobic methanotrophs since anaerobic methanotrophs seem to grow much slower (Krüger et al. 2008). This notion is supported by the fact that *Ca*. Methanoperedens were only detected in the forest KHs. Forest KHs have been indicated to be more prone to anoxic conditions due to lower photosynthesis via shading of surrounding trees and increased respiration as a consequence of increased allochthonous input of organic matter, e.g. leaf litter (Bizic et al. 2022). Agricultural KHs are more prone to frequent oxygen input by wind-induced mixing (Nitzsche et al. 2017). This may promote aerobic methanotrophs, at least at the sediment surface while lower sediment parts are likely fully anoxic. Generally, oxygen status is considered an important driver for methanotroph and methanogen abundances, e.g. different levels often favor different taxa of aerobic methanotrophs (Guerrero-Cruz et al. 2021). Since in our study, oxygen level could not be determined, a potential variation of oxygen levels between KHs or samples might be responsible for detected differences in both microbial abundances and methane production potential.

Contrastingly to *Ca*. Methanoperedens, the members of *Methylomirabilaceae* had high relative abundances in agricultural KHs and contributed up to half of all methanotroph ASVs. In contrast to aerobic methanotrophs, the relative abundance of *Methylomirabilaceae* showed no correlation to potential net CH_4_ production. Although *Ca*. Methylomirabilis was not detected, it is often assumed that members of the family *Methylomirabilaceae* are capable of oxidizing CH_4_ (Jiang et al. 2022, Yang et al. 2023). However, recent evidence for methylotrophic rather than methanotrophic capabilities in *Methylomirabilaceae* genomes, including *Ca*. Methylomirabilis (Rasmussen et al. 2024) challenges this concept. Therefore, the functional role of this group remains unclear, as there is currently little known about this bacterial group. Usually, the Sh765B−TzT−35 group occurs in deeper, anaerobic lake sediments (van Grinsven et al. 2022, Yang et al. 2023, Jiang et al. 2022).

Generally, our results prove that there is potential for AOM in KHs, but the true extent remains unknown as we did not directly measure anaerobic CH_4_ oxidation rates. The occurrence of *Ca*. Methanoperedens only in forest samples contradicts our hypothesized general promotion of anaerobic methanotrophs in agricultural KH sediments. The role of AOM in agricultural KHs should be further investigated, as our results did not directly confirm the presence of anaerobic methanotrophs, although they have been detected and isolated from agriculturally influenced waters and soils (Raghoebarsing et al. 2006, Ettwig et al. 2009 , Vaksmaa et al. 2017). However, an impact of land use on AOM is indicated by our results, either by promoting different taxa of anaerobic methanotrophs or AOM actually playing a lesser role in agricultural compared to forest KHs.

### Composition of the methane-cycling microbiota

The detected aerobic methanotrophs were all affiliated to the *Gammaproteobacteria*. Gammaproteobacterial methanotrophs are frequently found as the only or predominant CH_4_-oxidizing bacteria in low-oxygen or even anoxic freshwater environments such as lake water columns and sediments (Oswald et al. 2016). The predominance of *Crenothrix* in sediment samples of agricultural KHs adds to recent evidence for a high importance of this methanotroph in similar ecosystems. Its activity is important in shallow eutrophic lake sediments (Yang et al. 2022), and it can be a key methanotroph for the mitigation of CH_4_ emissions from lake water columns (Oswald et al. 2017). Our RDA revealed an association of *Crenothrix* with relative abundance of methanogens, high sulfate, and, to some extent, with neutral to slightly alkaline pH. These findings suggest an association with high inorganic solute loads. *Crenothrix* can easily adapt to fluctuating redox conditions (Frindte et al. 2016), which might be a useful trait for thriving in KH sediments surrounded by cropland, as they have to adapt to frequent but irregular oxygen inputs through higher wind exposure. Contrastingly, the genus *Methylobacter* (Gammaproteobacteria) was more abundant in the forest KH samples that are less exposed to wind thus also to water column mixing. This conclusion is supported by previous studies that observed *Methylobacter* under low oxygen or even anoxic conditions in deep lakes (Martinez-Cruz et al. 2017, He et al. 2012, Deutzmann et al. 2011). Therefore, the here observed strong association of *Methylobacter* with forest KHs and high SUVA in the RDA may be due to the likely higher prevalence of hypoxia in sediments of forest KHs.

The high proportion of hydrogenotrophic methanogens detected in all our samples indicates a high relevance for this methanogenic pathway in flooded KH sediments independently of the land use type. Hydrogenotrophic methanogenesis can be expected as typical in fresh water lake sediments, since the organic material cannot be completely degraded due to oxygen limitations and fermentation processes that yield CO_2_ and H_2_ are fostered (Conrad 2020). The low abundances of methylotrophic methanogens suggested a limited relevance for this pathway in KH sediments. Acetoclastic methanogens and thus acetoclastic methanogenesis seem to be selected by cropland as land use type.

Generally, methanogen diversity is expected to increase along with higher pH and inorganic solute concentrations (Bräuer et al. 2020). This is supported by our findings. Additionally, these parameters and further variables, i.e. sediment C and water content, significantly contributed to the explanation of the methanogen microbiota structure (Fig. 4). A higher variability of methanogen taxa among the agricultural KH samples is reflected by higher scattering as compared to forest KH samples (Fig. 3).

We conclude that land use type has a pronounced effect on the structure and biodiversity of both the general and especially the methane-cycling microbiota. In a previous study in the same region, geographical proximity of KHs does not correlate with physicochemical similarity or taxa distribution and no effect by land use on the general microbial diversity was observed (Ionescu et al. 2022). Instead, a biodiversity homogenization effect along with eutrophication was proposed. It should be noted that previous studies that did not find an effect of land use on microbiota were broader in scope and did not distinguish between different crops or tree species (Ionescu et al. 2022, Bizic et al. 2022). Thus, our study shows for the first time that there can be in differences the general microbiota correlated with land use. All of the here sampled KHs were surrounded by the same tree or crop species, except for KH agr2, making the land management effects more pronounced. Nonetheless, a general effect of land use on methanotrophs and methanogens relative abundance in KHs has been previously shown (Ionescu et al. 2022). Both were higher in grassland KHs with slight but insignificant differences between agricultural and forest KHs. As sediment and water samples were considered together for that analysis, this might have underestimated the true effect of land use on sediment methane-cycling microbiota, as we have seen in our study. Further literature about land use affecting water body microbiota has been scarce. In lakes, an indirect effect was shown (Marmen et al. 2020). In river sediments, manure runoff caused significant differences in microbial composition and promoted several genera including *Crenothrix* (Beattie et al. 2020), thus showing a similar effect to our study.

### Impacts on potential CH_4_ production from KHs

The lack of significant or even apparent difference by land use in addition to the high range of measured CH_4_ production in our samples suggests that the potential net CH_4_ production in the samples is controlled by more individual factors in the KHs. The parameters identified to be most relevant in the PLSR projection of CH_4_ production also showed much individuality between single KHs and were related to relative abundances of methane-cycling microbiota, inorganic solutes, water content, and TIC. The parameters with low impact (i.e. related to Shannon index, pH) were mostly among the ones that were significantly different between the land use groups, but not so much between individual KHs (Table 3). This was different for soil texture parameters. sand and clay, which were classified as moderately influential. Most WEOM property indices were also among the moderately influential variables, possibly reflecting their relation to substrate quality, which is important for methanogenesis (Conrad 2020). Nitrate concentration was ranked as highly influential, despite the generally low nitrate levels in the sampled KHs. Likely, these were caused by competing microbial processes such as denitrification and DNRA, which have been indicated as relevant for inundated KH zones before (Reverey et al. 2018). These also might explain the comparatively higher ammonium concentrations found in our samples.

The measured potential net production rates of CH_4_ in our study suggest that land use cannot serve as a proxy for higher or lower emissions, which might be in line with previously observed homogenization effects (Ionescu et al. 2022) and general eutrophication of KHs in the area (Lischeid et al. 2018). However, land use can clearly impact variables with high importance for projecting potential net CH_4_ production, such as relative abundances of methane-cycling microorganisms, as shown in our study, and inorganic solute levels including sulfate and nitrate (Ionescu et al. 2022, Nitzsche et al. 2017). The impact of land use on relative abundances of methane-cycling microorganisms but not on net methane production is likely explained by different activities of these microbes in the samples, which were not determined in our study and did not result in land use-specific differences in net CH_4_ production.

As we could only measure potential greenhouse production rates, solid estimates of on-site CH_4_ emission and its relationship to environmental KH variables are difficult to be drawn from our results. In the natural setting, both methanogen activities from deeper sediment parts as well as methanotrophs in the water column (Dean et al. 2018) may impact *in situ* CH_4_ emissions from KHs that remain unexplored in this study. Nevertheless, we sampled in our study the most active, i.e. upper part of the KH sediments. There is a pronounced seasonality and even legacy effect on KH GHG production and on sediment microbiota by changing temperature, nutrient levels, and sediment water content along with water table changes (Reverey et al. 2018, 2021). In our study, no water tables have been recorded before the sampling. However, it can be assumed that as they were flooded at the time point of sampling (July 2017), they likely had been all year as KHs of similar type record the lowest water levels in late summer or autumn (Kazanjian et al. 2018, Reverey et al. 2018). Thus, potential legacy effects of dry–wet cycles in previous years remain unexplored, as we have targeted only flooded sediments, which are characterized by anaerobic processes such as CH_4_ production and fermentations (Attermeyer et al. 2017). Similarly, our sampling only targets one specific timepoint in summer, while many environmental parameters in KHs have been shown to fluctuate throughout the year, mostly those related to ion and inorganic solute concentrations as well as redox parameters (Lischeid et al. 2018, Ionescu et al. 2022). These temporal changes may impact on microbes and thus also on methane emissions. However, our sampling was performed on a timepoint that ensured both flooded, i. e. anaerobic or low oxygen conditions within the KH as well as high temperatures, which are considered main drivers for methanogenesis (Saunois et al. 2020). Therefore, our study likely captures the most relevant seasonal and spatial part of the KHs in regard to CH_4_ production, but legacy and seasonal effects that might play a role in observed differences in microbiota composition, the dynamics of functional groups and/or net CH_4_ production potentials between individual KHs were not considered. Thus, we cannot fully explain observed differences between KH’s datasets. This is a major outcome of our study and calls for *in situ* studies more comprehensively capturing temporal CH_4_ greenhouse gas production dynamics and associated microbiota dynamics to better understand responses of sediment microbiota, in particular those of methanogens and methanotrophs, to changes in determinative environmental variables.

## Conclusion

Our study identified significant differences in various microbiota characteristics of inundated KH sediments dependent on the surrounding land use type. Similarly, many sediment variables were different between KHs with agriculture or forest land use, however, many also varied between single KHs within the same land use type. Especially inorganic solute concentrations (sulfate, nitrate, and ammonium) that can have an effect on methanotroph and methanogen microbiota composition and activity might have led to different potential net CH_4_ and CO_2_ production rates as observed between individual KHs. Eventually, our results suggest that land use type seems to be a neglectable proxy for CH_4_ emission potentials in KHs inundated sediments. However, fluctuations in water table, temperature, and nutrient load throughout the year influence CH_4_-cycling microbiota composition, abundance, activity, and interactions, and thus, eventually CH_4_ emission patterns and fluxes. Therefore, a seasonal impact that was not covered by the current sampling and study design may occur and requires further studies that target seasonal variations of CH_4_ emissions and microbiota components involved.

## Supplementary Material

fiaf050_Supplemental_Files
