# Supplementary material for: Detectable land use impact on methanotrophs and methanogens in kettle hole sediments but not on net methane production potentials
Source: FEMS Microbiol Ecol. 2025 May 1;101(6):fiaf050. doi: 10.1093/femsec/fiaf050 (PMC12089754; doi:10.1093/femsec/fiaf050)
Supplement: fiaf050_Supplemental_Files [file fiaf050_supplemental_files.zip › Supplementary data_figures_revision.docx]

**Supplementary Material**

**Detectable land use impact on methanotrophs and methanogens in kettle hole sediments but not on net methane production potentials**

Danica Kynast, Florian Reverey, Lars Ganzert, Hans-Peter Grossart, Gunnar Lischeid and Steffen Kolb


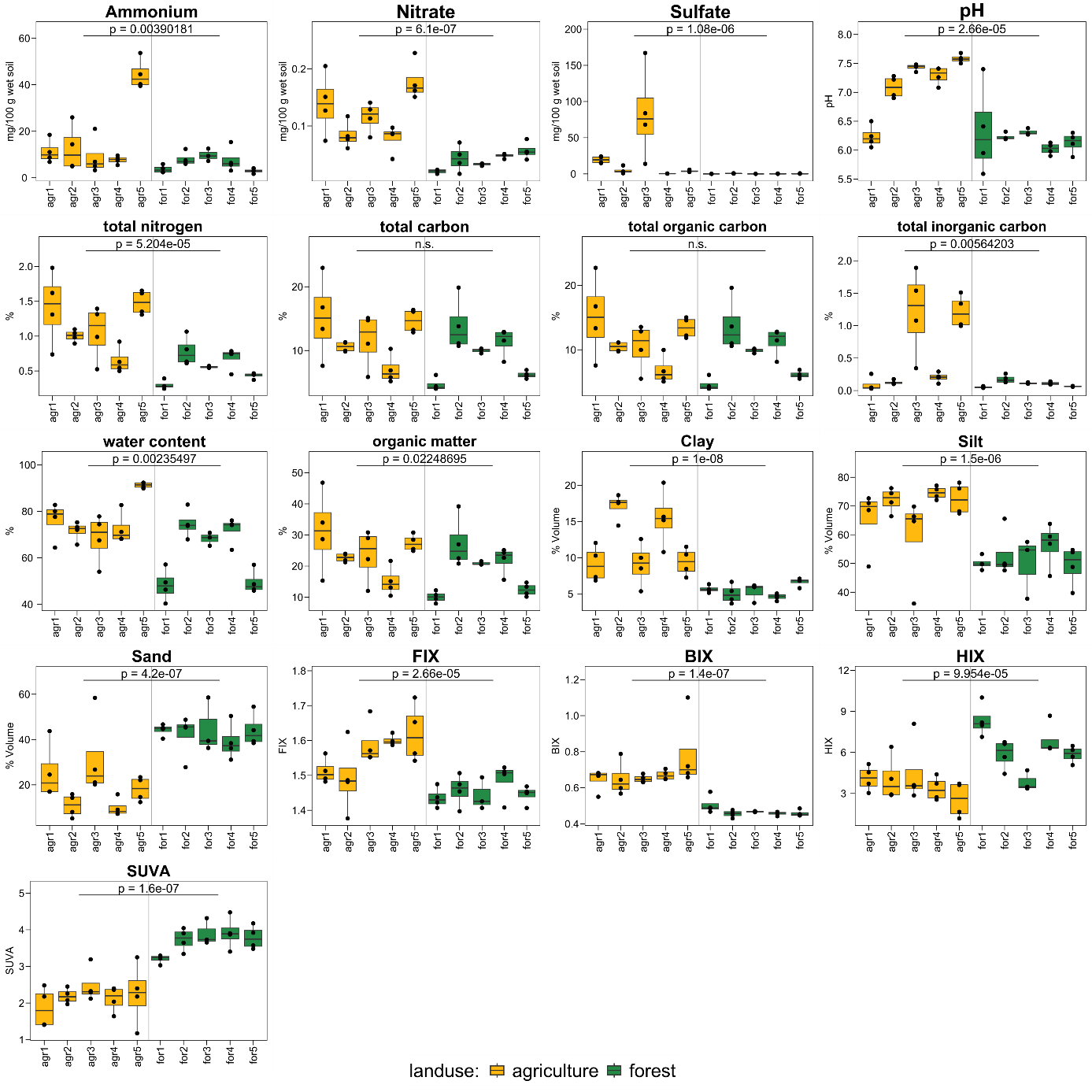


**Figure S1**: Boxplots of soil parameters for each sampled kettle hole. Land use types are indicated by color. Statistical results (p values) of a Wilcoxon test comparison between land use group are given on top of the plot (n.s., not significant).


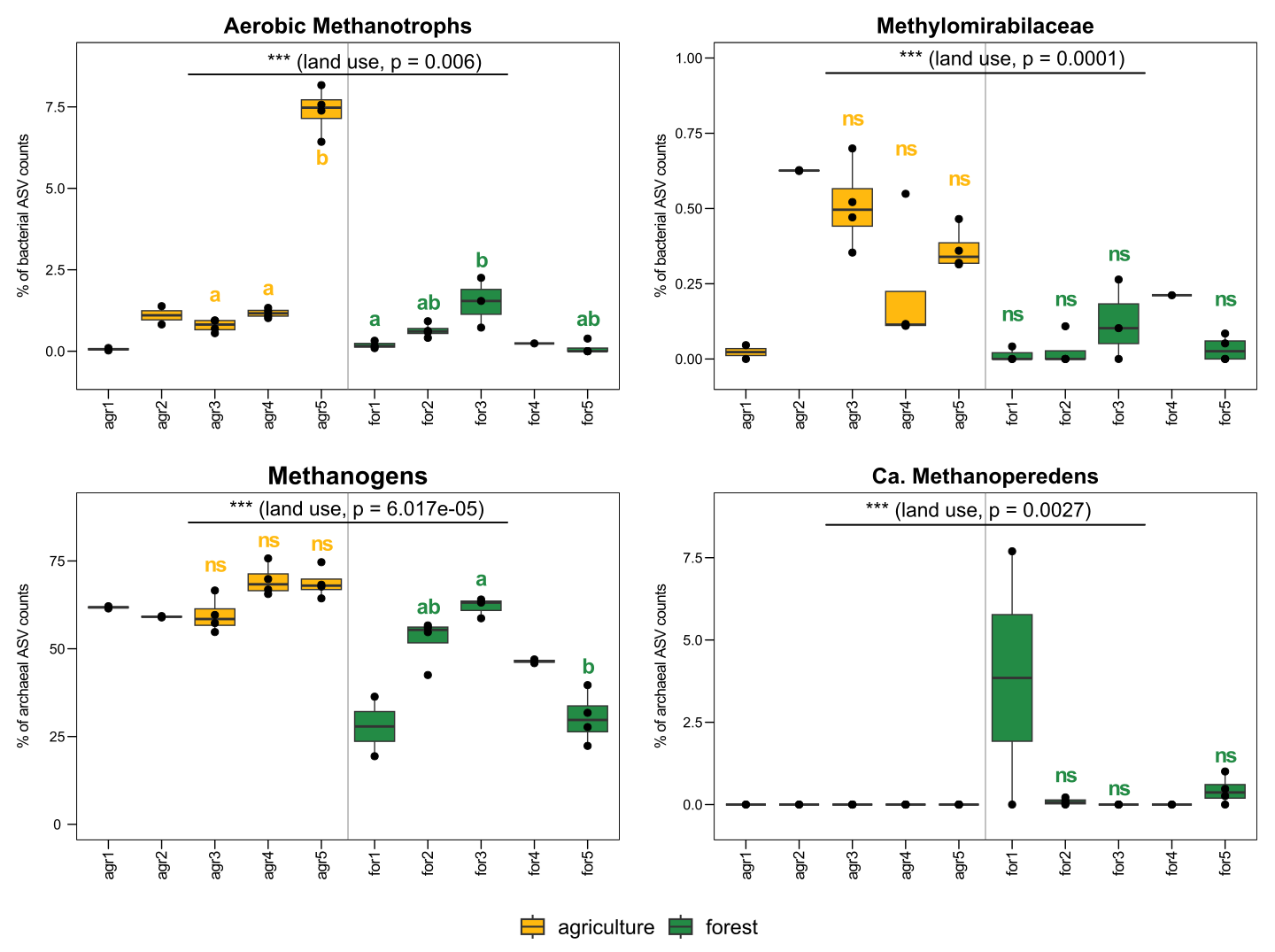


**Figure S2**: Relative abundances of aerobic methanotrophs, Methylomirabiliceae family members, methanogens and Ca. Methanoperedens expressed as percentages of total ASV counts of the total 16S rRNA sequences of bacteria or archaea, respectively, in sediments from the respective kettle holes (KH). Surrounding land use of the KHs is indicated by color and identifier of the KH (x-axis). Significant differences between kettle holes of the same land use of adjusted p values of <0.05 are indicated by letters of the respective color (Kruskal-Wallace test followed by Dunn’s test; only KHs with data for at least three samples were considered). Significant differences between samples are represented by contrasting labels (“a” and “b”). Significances between samples grouped by land use are indicated in black letters on top of the plot (Wilcoxon-test; all available samples were considered). Non-significances are indicated with “ns”.


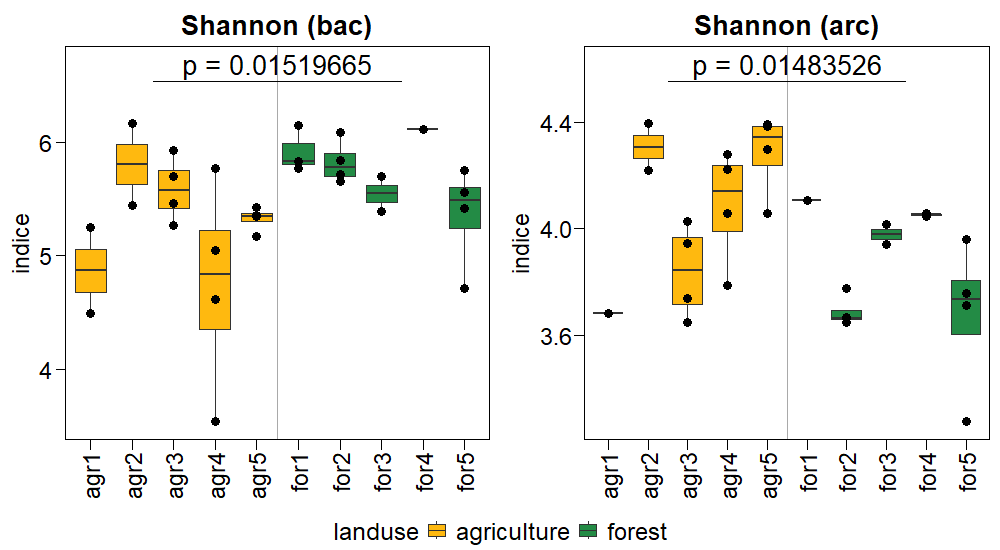


**Figure S3**: Shannon alpha diversity indices for bacteria (bac; left) and archaea (arc; right). The indices were calculated from the respective 16S rRNA gene ASV table for bacteria and archaea. Land use is indicated by color and significant differences by land use type is indicates as p values on top of the plot (Wilcoxon-test).


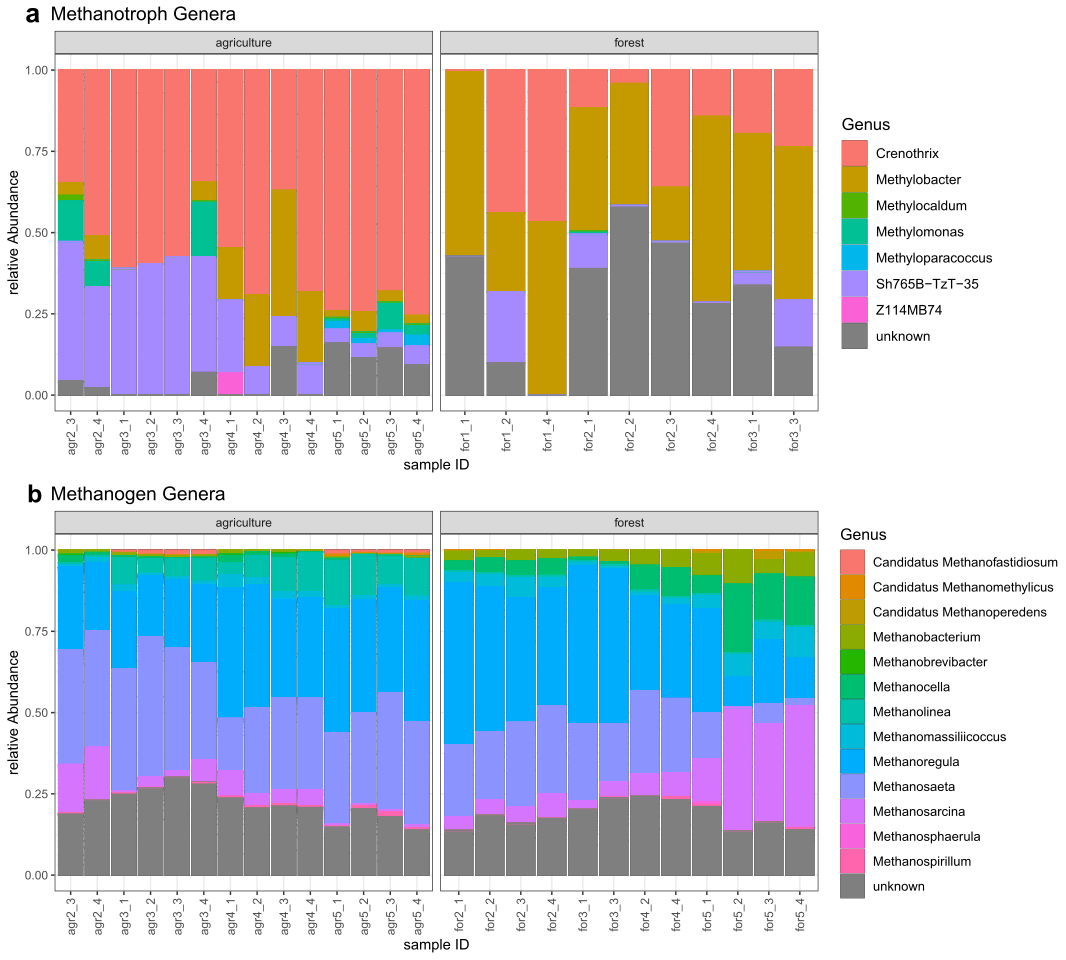


**Figure S4**: Relative abundances of genera of methanotrophs (a) and methanogens (b) detected in kettle hole sediment samples. Genera were determined by 16S rRNA gene sequencing for bacteria and archaea, respectively, and are indicated by color.


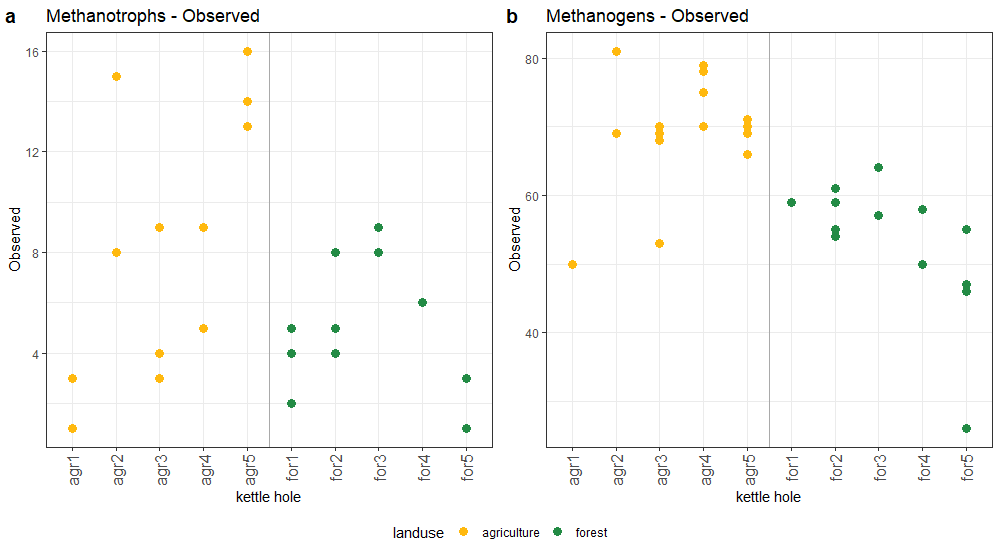


**Figure S5**: Observed richness of methanotrophs (a) and methanogens (b) in kettle hole sediments from surroundings of two different land uses (color of points). Note that the here depicted samples for methanotrophs include all samples with sufficient quality where any amount of methanotrophs were detected, but for later analyses some samples had to be removed due to very low total ASV counts.
